# The clinicopathologic features and response to treatment of patients with Nonhodgkin Lymphoma: A single-center experiment in Turkey

**DOI:** 10.12669/pjms.35.1.415

**Published:** 2019

**Authors:** Rahsan Yildirim, Gulden Sincan

**Affiliations:** 1 Dr. Rahsan Yildirim, Department of Hematology, Medical School, Ataturk University, Erzurum, Turkey; 2 Dr. Gulden Sincan, Department of Hematology, Erzurum Regional Training and Research Hospital, Erzurum, Turkey

**Keywords:** Nonhodgkin lymphoma, Laboratory findings, Response to treatment

## Abstract

**Objective:**

We aimed to compare laboratory features, histopathological types, response to treatment of patients with non hodgkin lymphoma in our department and other regions.

**Methods:**

A total of 80 patients nonhodgkin lymphoma were evaluated. Because we had only 80 patients with complete data, we used T test for comparison of groups. We evaluated the parameters affecting surveillance with cox regression analysis.

**Results:**

The most common histological types of nonhodgkins lymphoma was diffuse large b cell lymphoma (n: 63, 78.75%). Thirty-nine percent of all patients had anemia, 32% had hypoalbunemia, 71.25% had elevated serum LDH, 32.5% had elevated serum ß2 microglobulin value. Advanced age, the presence of bulky disease, elevated Ki-67 level, IPI score, refractory to first line treatment were found to be correlated with shorter survival time. We treated 77 (96.25%) patients with doxorubicin containing regimen. Complete and partial remission rates of first line treatment were 77.5% and 10%, respectively. Seven (8.75%) patients died because of disease progression and 1 (1.25%) patient died due to sepsis.

**Conclusion:**

The frequency of lymphoma subtypes, clinical characteristics, treatment outcomes and survival rate vary from region to region. Therefore it is important to determine dissimilarity of these parameters for improve of survey.

## INTRODUCTION

Non-hodgkin lymphoma’s (NHL) are a heterogeneous group of lymphoproliferative malignancies.^1^ Almost 85% of NHLs are of B-cell origin; only 15% are derived from T/NK cells. Based on the World Health Organization (WHO) classification, the two most common NHL’s subtypes are diffuse large B-cell lymphoma (DLBCL), occurring in 31% of patients, and follicular lymphoma (FL), occurring in 22% patients.

The prognosis and type of treatment of NHL depend on the histological subtype, disease stage and associated comorbid conditions. Chemotherapy and radiotherapy are the two principal forms of treatment. Autologous and allogenic stem cell transplantations and a watch-and-wait strategy are also used to treatment some NHL subtypes. CAR T cells, blinatumumab, immune checkpoint inhibitors are novel drugs used for treatment of NHL.

## METHODS

We examined 80 patients with NHL retrospectively. Approval of the Ataturk University Ethics Committee was obtained for the study. Excision biopsy was performed for diagnosis in most of the cases. A diagnosis of NHL was established according to WHO criteria. Complete blood count, albumine, lactate dehydrogenase, uric acid, ferritin, beta 2 (ß2) microglobuline levels of all participants were determined from patients records. B symptoms were defined as > 38°C fever, night sweating, > 10% weight loss in the last six months. Ann Arbor classification was used for staging of patients. Stage of disease, presence of bulky disease, extranodal involvement, performance status of Eastern Cooperative Oncology Group (ECOG), score of international prognostic index (IPI), type of treatment applied and response to treatment of all patients were determined. The evaluation of treatment response was made in accordance with WHO criteria. Overall survival (OS) was defined as time from start of treatment to either death from any reason. All statistical analyses were performed using SPSS version 20.0. Numerical data were expressed as number and percentage. We used T test for comparison of groups. Cox regression analysis was used for survey assessment. P<0.05 was considered statistically significant.

## RESULTS

In this study, 80 patients with NHL were evaluated. Thirty three patients were females and 47 males (male/female ratio: 47/33 = 1.4), and the median age was 57.6±16.6 years (range: 18-87 years). There wasn’t statistically significant difference in age between male and female group (p value: 0.01). B-cell and T-cell originated NHL were identified in 76 (95%) patients and in 4 (5%) patients, respectively. DLBCL was the most commonly histopathologic type (78.75%). The most common subtype of T-cell lymphomas was the peripheral t cell lymphoma (NOS) with 3 patients. Histopathological classification of the all patients is showed in Table-I. Comorbid diseases are specified in Table-II.

**Table-I:** Distribution of histological subtypes in patients with lymphoma.

Subtypes of NHL	Number of Cases	Percent (%)
Diffuse Large B Cell Lymphoma	63	78.75
Peripheral T Cell Lymphoma	4	5
Follicular Lymphoma	4	5
Mantle Cell Lymphoma	3	3.75
Burkitt Lymphoma	2	2.5
Marginal Zone Lymphoma	2	2.5
Small Lymphocyctic Lymhoma	2	2.5

**Table-II:** Comorbid Disease Associated With Non-Hodgkin Lymphoma.

Disease	Number of Patients	Percent (%)
Hypertension	10	12.5
Diabetes Mellitus	5	6.2
Goiter	5	6.2
Chronic Obstructive Pulmonary Disease	3	3.8
Rheumatoid arthritis	2	2.5
Benign Prostatic Hyperplasia	2	2.5
Coronary Artery Disease	2	2.5
Chronic hepatitis	1	1.25
Hyperlipidemia	1	1.25
Gastric Cancer	1	1.25
Invasive Ductal Cancer	1	1.25

According to Ann Arbor Staging System, 7.5% (n:6) of all patients had Stage I, 31.25% (n:25) had Stage II, 16.25% (n: 23) had stage III, 32.5% (n: 26) had Stage IV. B symptoms were seen in 45 (56.25%) patients. B symptoms were seen more common in patients with ≥ 60 ages rather than in patients with <60 ages (p<0.01). ECOG performance status was stage 1 in 11 (13.75%) patients, was stage 2 in 37 (46.25%) patients, was stage 3 in 26 (32.5%) patients and was stage 4 (7.5%) in 6 patients. The presence B symptoms, elevated Ki-67 level, advanced stage, extra lymphatic involvement were associated with high ECOG score, significantly. IPI score was 0 in 1 (%) patients, was 1 in 8 (%), 2 in 16 (%), 3 in 32 (%), 4 in 19 (%), and 5 in 4 (%) patients at the time of diagnosis. 12 (15%) patients had bulky mass. Bulky disease was seen more common in patients who had comorbid disease (p<0.02). Extranodal involvement was determined in 36 (45%) cases. The most frequently sites of extranodal involvement were: gastrointestinal system (n:12, 15%), lung (n:7, 8.75%), spleen (n:6, 7.5%), bone (n:5, 6.25%), liver (n:4, 5%), renal (n:2, 2.5%).

Laboratory findings were as follows; the levels of hemoglobine was 11.6±2.6 (min:6 max.16.1), white blood cell (WBC) was 27095±12620 (min:11000, max:27050), uric acid was 7.5±2.2 (min:3.7, max:12.4), ß2 microglobuline was 4.6±3 (min:2, max.16), ferritin was 385.5±668.9 (min: 8, max.4400), Ki-67 76±14.1 (min:30, max:95). Thirty nine patients had anemia, 11 had leucostosis, 57 had elevated LDH level, 32 had hypoalbunemia, 26 had elevated ß2 microglobulin, 32 had elevated uric acid level.

We treated 71 (88.75%) patients with R-CHOP, 6 (7.5%) patients with CHOP, 2 (2.5%) patients with R-Hypercvad and 1 (1.25%) patient with R-Gemox treatment protocols for first line treatment. Sixty two (77.5%) patients achieved a complete response, 8 (10%) patients had a partial response with first line therapy. Four (5%) patients had stable disease and 6 (7.5%) patients had progressive disease. The median time to first relapse after the first line treatment was 26 months. In second-line treatment, R-ICE regimen was used in 18 (22.5%) patients, R-DHAP regimen was used in 10 (12.5%) patients although each of ICE regimen and DHAP regimen were used only one patient. Sixteen (20%) patients were achieved to complete remission with second line treatment. Each of partial remission and progressive disease were in 7 (8.8%) patients. Eight (10%) patients were treated with autologous bone marrow transplantation after salvage chemotherapy. We treated 3 patients with ASHAP regimen in third line treatment. seven (8.75%) patients died because of disease progression and one (1.25%) patient died due to sepsis. Mean survey was associated with advanced age, bulky disease, elevated ki-67 level, IPI score, response to treatment first line treatment. Mean survey time was 27.5±17.3 (min:1, max.60) months.

## DISCUSSION

NHL is the 6th most common type of cancer.^2^ The frequency of NHL subtypes varies according to geographical regions.^3^ Eighty- eighty five percent of NHLs are derivated from B-cells, while 15-20% are from T-cells in USA. In our study, B-cell origin lymphomas were found as 95%, and T-cell origin lymphomas as 5%. In our country, T cell originated lymphoma is very rare. The most common histological subtype of NHL is DLBCL with a frequency of 31%. DLBCL is very common (78.75%) in our country compared to USA, Asian and European countries. FL is rare in Chine and Middle East while it is more common in USA. We found that follicular lymphoma incidence was 5% and it was rare in our country. This may be explained by follicular lymphoma was relatively less because of DLBCL had very higher incidence in our country.

NHL is more common in people aged over 65 year in U.S.A. The median age was 57.6 in our study and NHL was seen at an earlier age in our country rather than U.S.A. Isikdogan et al reported that the median age was 43 years in patients living in the southeast Turkey.^4^ In addition, the average age of at diagnosis was 44 years in one study that was made by Barista et al. in central Turkey.^5^ The median age of our patients was higher than the patients in southern and central Turkey. The incidence of lymphoma is higher in men than in women. In U.S.A and Europa, male dominancy rates is different.^6^ Male/female ratio was 1.4 in our study according to literature. It was reported that NHL was seen in men at an earlier age. But in our study, it wasn’t significantly difference between men and women in terms of age (p value: 0.06).

The current staging system for NHL in adults is known as the Lugano classification, which is based on the older Ann Arbor System.^7^ Most patients with non-Hodgkin’s lymphomas had advanced stage.^8^ In our study; stage 3 and 4 NHL was found in 49 patients according to literature. The effects of the presence of B-symptoms on the survival rate have been reported in a study by Maksymiuk et al.^9^ In our study, we didn’t find a relationship between the presence of B symptom and survey time (p: 0.06). The ECOG scale ≥2 was an adverse prognostic factor.^10^ We didn’t determine the relationship with ECOG score and survey time (p: 0.06).

The extranodal involvement was observed in 20-30% of patients with NHL.^10^ In a study performed by Tabakan et al. in Turkey, extranodal involvement was detected in 43.9%.^11^ The extranodal involvement was found as 44% in the study by Isikdogan.^4^ We found an extranodal involvement ratio of 45% according to the studies that were made in Turkey. The most common site of extranodal involvement was the gastrointestinal system with 15% in our study. The incidence of gastric involvement was 66% in a study in Ankara by made Alican et al.^12^ We found that the gastric involvement incidence was 12.5%. A correlation between survey time and extranodal organ involvement has not been reported in the literature.^13^ We didn’t detect that the effect of extranodal involvement on survey time (p:0.07).

In the literature, lower serum albumin levels adversely effected the survival time.^14^ In our study a statistically significant difference was not found between serum albumin level and survival time (p:0.06). It was detected that anemia was associated with poorer CR rate.^10^ We didn’t find any correlation with anemia and survey time (p: 0.07).

The nuclear proliferation antigen Ki-67 is a marker of tumor cell proliferation rate. Higher Ki-67 level was associated with poorer survival.^15^ Miller and et al evaluated that the effect of high Ki 67 (Ki-67 ≥80%) on survive time. They didn’t find significantly relationship between Ki-67 level and survey time.^16^ We considered >60% Ki-67 value as high. We detected that the mean survey time was shorter in the patients that had high Ki-67 value (p: 0.01).

Patients with NHL who is treated with doxorubicine containing regimen have high complete remission rate. Rate of complete response to treatment is around 60-80% in NHL. Five year survival rate is over 55% and it was reported as 54.6% in European study.^17^ Five-year survival rate was reported to range 50-60% in Scandinavian countries.^18^ In our study, 5-year survival rate was 45% and it was lower in our country than Europa.

There were 63 patients with DLBCL in our study and the rate of response to first line treatment was 71.4%. It was reported that the remission rate was 65-70% with anthracycline-based regimens plus anti-CD20 antibody in patients with DLBCL.^19^ The response to first line treatment in patients with DLBCL was according to literature.

## CONCLUSION

The frequency of NHL subtypes varies from region to region. There are differences between regions in terms of clinicopathological features and response to treatment. Therefore it is important to identify these differences for management of patients.

### Authors’ Contributions

**GS:** Designed and did statistical analysis, manuscript writing

**RY:** Did data collection.
